# The Redox Paradox: Cancer’s Double-Edged Sword for Malignancy and Therapy

**DOI:** 10.3390/antiox14101187

**Published:** 2025-09-28

**Authors:** Jyotsna Suresh Ranbhise, Manish Kumar Singh, Songhyun Ju, Sunhee Han, Hyeong Rok Yun, Sung Soo Kim, Insug Kang

**Affiliations:** 1 Department of Biochemistry and Molecular Biology, School of Medicine, Kyung Hee University, Seoul 02447, Republic of Korea; jogm25@khu.ac.kr (J.S.R.); manishbiochem@gmail.com (M.K.S.); thdgus8543@khu.ac.kr (S.J.); sunheehan@khu.ac.kr (S.H.); foryou018@naver.com (H.R.Y.); 2 Biomedical Science Institute, Kyung Hee University, Seoul 02447, Republic of Korea; 3 Department of Biomedical Science, Graduate School, Kyung Hee University, Seoul 02447, Republic of Korea

**Keywords:** reactive oxygen species (ROS), oxidative stress, cancer, redox signaling, Nrf2, glutathione (GSH), thioredoxin (Trx), cancer therapy, tumor microenvironment (TME), ferroptosis, PI3K/AKT/Mtor, PTEN

## Abstract

Reactive oxygen species (ROS) function as critical signaling molecules in cancer biology, promoting proliferation, angiogenesis, and metastasis at controlled levels while inducing lethal damage when exceeding the cell’s buffering capacity. To survive under this state of chronic oxidative stress, cancer cells become dependent on a hyperactive antioxidant shield, primarily orchestrated by the Nrf2, glutathione (GSH), and thioredoxin (Trx) systems. These defenses maintain redox homeostasis and sustain oncogenic signaling, notably through the oxidative inactivation of tumor-suppressor phosphatases, such as PTEN, which drives the PI3K/AKT/mTOR pathway. Targeting this addiction to a rewired redox state has emerged as a compelling therapeutic strategy. Pro-oxidant therapies aim to overwhelm cellular defenses, with agents like high-dose vitamin C and arsenic trioxide (ATO) showing significant tumor-selective toxicity. Inhibiting the master regulator Nrf2 with compounds such as Brusatol or ML385 disrupts the core antioxidant response. Disruption of the GSH system by inhibiting cysteine uptake with sulfasalazine or erastin potently induces ferroptosis, a non-apoptotic cell death driven by lipid peroxidation. Furthermore, the thioredoxin system is targeted by the repurposed drug auranofin, which irreversibly inhibits thioredoxin reductase (TrxR). Extensive preclinical data and ongoing clinical trials support the concept that this reliance on redox adaptation is a cancer-selective vulnerability. Moreover, novel therapeutic strategies, including the expanding field of redox-active metal complexes, such as manganese porphyrins, which strategically leverage the differential redox state of normal versus cancer cells through both pro-oxidant and indirect Nrf2-mediated antioxidative mechanisms (triggered by Keap1 oxidation), with several agents currently in advanced clinical trials, have also been discussed. Essentially, pharmacologically tipping the redox balance beyond the threshold of tolerance offers a rational and powerful approach to eliminate malignant cells, defining a novel frontier for targeted cancer therapy.

## 1. Introduction

Cellular life operates on a delicate balance of chemical reactions, chief among them being redox processes [1]. Redox (oxidation-reduction) reactions involve electron transfer reactions between chemical species and are fundamental processes in all living organisms, participating in numerous biological cellular functions in aging, diseases, stress, and metabolism [2,3]. But, for a long time, the byproducts of redox reactions, ROS and reactive nitrogen species, were thought to cause damaging effects exclusively [4]. This perspective rapidly gave rise to the oxidative stress paradigm, a phenomenon caused by an overabundance of ROS overwhelming the cell’s ability to detoxify these reactive products, leading to indiscriminate damage to lipids, proteins, and DNA [5]. However, this damaging perspective of ROS has evolved significantly. In the past few decades, a trend for the appreciation of reactive species for their role in many signaling pathways has increased [6]. In normal physiological conditions, cells carefully maintain optimal concentration and distribution of intracellular reactive species, supporting necessary signaling pathways like “redox signaling” [7]. This dual nature of ROS is dramatically exploited in the context of cancer than anywhere else [1,8]. This brings us to a central concept in modern biology: the Redox paradox. In cancer cells, heightened ROS levels act as pro-tumorigenic factors [7,9], including increased glucose metabolism, adaptation to hypoxic environments, and oncogenic mutations [10]. However, the toxic level of ROS production also has a beneficial outcome; they are anti-tumorigenic, causing an increased level of oxidative stress and induction of tumor cell death [11]. For this reason, therapies involving ROS production, either to eliminate or elevate, may be promising in cancer therapy. This complex interplay, where ROS simultaneously drives and can be used to combat malignancy, defines the ‘Redox Paradox’ as a critical challenge and a novel approach to therapeutics-resistant cancer [12]. For this reason, therapies involving ROS production, either to eliminate or elevate, may be promising in cancer therapy.

## 2. Architects of the Malignant Redox State

### 2.1. ROS Sources

The malignant reprogramming by cancer cells to rewire their entire redox is a two-part process. First, cancer cells dramatically increase their endogenous ROS production through oncogenic signaling [13]. Second, to survive this self-inflicted oxidative stress, they must simultaneously build a powerful and hyperactive antioxidant defense system. ROS are reactive radicals or non-radicals generated from partial molecular oxygen metabolism [14]. Among them, free radicals contain one unpaired valence electron in their outer shell, making them highly reactive and unstable [15]. Widely known ROS include superoxide anion (O_2_^•−^), hydroxyl radical (·OH), hydrogen peroxide (H_2_O_2_), nitric oxide (·NO), and hypochlorous acid [16]. The O_2_^•−^ is short-lived, local, and does not cross the cellular membrane easily, generated from mitochondrial complexes 1, 2, and 3 [17,18]. The cytosolic O_2_^•−^ is rapidly converted to H_2_O_2_ by the enzymatic activity of superoxide dismutase 1 (SOD1) [19]. In mammalian cells, there are 41 sources of O_2_^•−^ and H_2_O_2_-producing enzymes [20] and in cancer, O_2_^•−^ and H_2_O_2_ are the most well-studied ROS, whereas H_2_O_2_ is the best-described ROS signaling molecule [10]. Hence, these elevated and often dysregulated levels of H_2_O_2_ in the tumor microenvironment represent a critical redox vulnerability, making cancer cells susceptible to therapeutic strategies that leverage H_2_O_2_-driven oxidative stress, such as those employing redox-active metal complexes.

Mitochondria are central to cellular bioenergetics and, by extension, are an unintentional yet critical endogenous source of ROS [21]. During oxidative phosphorylation (OXPHOS), mitochondria generate approximately 90% of a cell’s energy through the electron transport chain (ETC), serving as an indispensable channel for energy metabolism and cell survival [22]. This entire process involves redox reactions that continuously generate and consume high-energy molecules, such as NAD^+^, NADP^+^, and FAD^+^, to generate adenosine triphosphate (ATP) and reduce molecular oxygen (O_2_) to water in the ETC [23]. Electron leakage from ETC complex 1 (nicotinamide adenine dinucleotide (NADH): ubiquinone (Q) oxidoreductase) and complex 3 (ubiquinol–cytochrome c reductase) are the two prime sites for O_2_^•−^ production [24]. Additionally, a small portion of O_2_ consumed in the ETC is continuously reduced by a single step of one-electron reduction, also releasing some O_2_^•−^ [25].

While this basal ROS production is a normal physiological byproduct, this scenario dramatically changes in the case of carcinomas. In cancer cells, ROS levels increase due to heightened metabolic activity and higher ATP demand, which facilitates rapid proliferation [26]. Cancer cells, despite possessing a fully functional mitochondria and adequate molecular oxygen, oxidize glucose to lactic acid, i.e., anaerobic respiration. This phenomenon, known as the Warburg effect, yields far less ATP than in OXPHOS in mitochondria, yet cancer cells manage to proliferate and grow rapidly [25]. Cancer cells’ mitochondria also rapidly generate ATP and aid in cell proliferation, and this high stress of energy production on the cell opens more paths for ROS generation and eventually ROS stress [27].

The next more important source of endogenous ROS is via an integral membrane enzyme family, NADPH Oxidases (NOXs) [28]. The NOX catalyze the reduction of O_2_ to O_2_^•−^, coupled to the oxidation of NADPH, and this enzyme family includes NOX1, NOX2, NOX3, NOX4, NOX5, and the dual oxidases Duox1 and Duox2 [29,30]. The generated O_2_^•−^ is rapidly converted to H_2_O_2_ by the action of the antioxidant enzyme, superoxide dismutase (SOD), by binding O_2_^•−^ to the active site of SOD, thereby transferring an electron to the SOD metal cofactor, reducing it. This transfer disrupts the bonds between the metal cofactor and nearby histidine, causing protonation of histidine and facilitating the release of molecular O_2_ as the first product. In the second half of the enzyme reaction, a new O_2_^•−^ binds to the SOD active site, receiving an electron from the previously reduced metal cofactor. This electron transfer promotes protonation of the new O_2_^•−^, ultimately generating H_2_O_2_ [28,31]. Interestingly, NOX-derived ROS and mitochondrial ROS amplify each other in a positive feedback loop. NOX-derived ROS increases mitochondrial ROS, and mitochondrial ROS stimulates NOX activation [32]. Research also suggests that NOX-dependent ROS generation is linked with oncogenic signaling of RAS and various other growth factors [33].

The endoplasmic reticulum (ER), a fine network of tubules, also contributes to ROS production in an eukaryotic cell. Apart from secretory pathways, the ER is also responsible for protein folding, biosynthesis, translocation, and post-translational modifications, including glycosylation, disulfide bond formation, and chaperone-mediated protein folding processes [34,35]. Evidence suggests that ER under stress undergoes protein misfolding, producing ROS, leading to oxidative stress [36]. ER also generates ROS, especially H_2_O_2_, while reoxidation of PDI active sites in the ER-associated degradation pathway [37]. Several NOXs are positioned in the ER membrane, catalyzing the generation of ROS. For instance, NOX4 produces H_2_O_2_ [38]. Increased ROS is also capable of causing ER stress and initiating the unfolded protein response [39]. Another major function ER plays is protein stabilization through the oxidative protein folding (OPF) reactions [40], O_2_ acts as a source of oxidizing equivalents necessary in intramolecular disulfide bond formation. This OPF is the major source of H_2_O_2_ [41].

**Figure 1:**
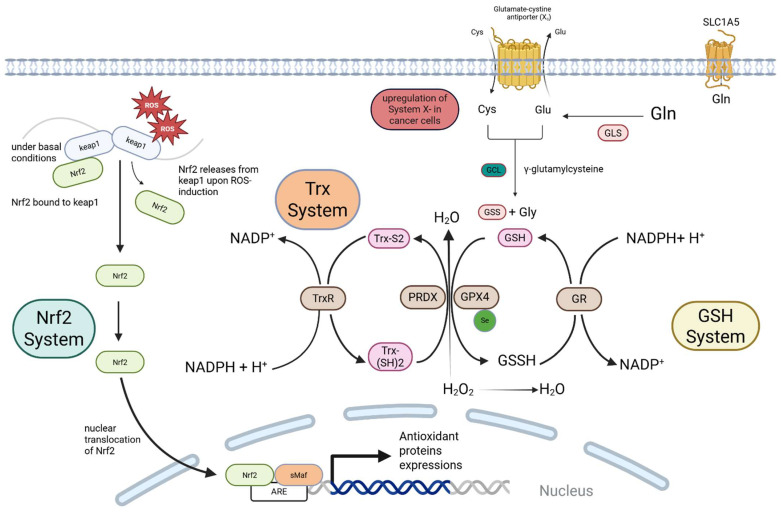
The Antioxidant Systems in Cancer Cells. Cancer cells exhibit a hyperactive antioxidant defense system orchestrated by the Nrf2, Glutathione (GSH), and Thioredoxin (Trx) pathways. Nrf2 Activation: High basal ROS triggers the release of the transcription factor Nrf2 from its inhibitor, Keap1. Nrf2 translocates to the nucleus, binds to the Antioxidant Response Element (ARE), and drives the expression of numerous antioxidant proteins. The GSH and Trx Systems: The GSH system, synthesized from glutamate, cysteine, and glycine, is the major cellular antioxidant. It detoxifies peroxides via enzymes like Glutathione Peroxidase 4 (GPX4). The Trx system, centered on Thioredoxin (Trx) and Thioredoxin Reductase (TrxR), reduces oxidized proteins and regenerates peroxiredoxins (PRDXs). Both systems are critically dependent on NADPH for the regeneration of their active forms (GSH and reduced Trx). Nrf2 activation transcriptionally upregulates key components of both systems, creating a robust, interconnected shield against oxidative stress.

Taken together, the combined ROS output from dysfunctional mitochondria, hyperactive NOX enzymes, and a stressed ER creates an immense oxidative intracellular environment. For a normal cell, such a massive and sustained ROS burden would be unsustainable, triggering apoptosis or senescence [42]. However, cancer cells adapt. To not only survive but thrive amidst this self-inflicted oxidative onslaught, they re-engineer their defensive capabilities by constructing a powerful and interconnected antioxidant shield (Figure 1) [43]. Leading direct hyperactivation of the master antioxidant regulatory systems, which are co-opted from cellular components into key enablers of malignancy [44]. This reliance on a finely tuned yet precarious redox balance also exposes vulnerabilities, offering opportunities for therapeutic intervention through exogenous agents, including redox-active metal complexes, that can tip the scales of oxidative stress back towards cytotoxicity in cancer cells.

### 2.2. Antioxidant Defense of Cancer Cells

#### 2.2.1. The Nrf2-Keap1 Axis

The cornerstone of the cellular antioxidant response is the Nrf2-Keap1 signaling axis [45]. Nuclear factor erythroid 2-related factor 2 (Nrf2) is a master regulator of various cytoprotective genes and pathways, such as glutathione synthesis, ROS scavenging, drug detoxification, and NADP synthesis [46]. Under normal, homeostatic conditions, Nrf2 is held inactive in the cytoplasm by its negative regulator, Kelch-like ECH-associated protein 1 (Keap1). Keap1 acts as a sensor for oxidative stress, binding to Nrf2’s Neh2 domain and targeting it for constant degradation by 26S proteasome, thus ensuring that the free Nrf2 in the cell is at appropriately low levels [47,48]. Hinge and latch theory suggests that when the cell is exposed to oxidative or electrophilic stress, cysteine residues in the IVR region on Keap1 dimer are modified. This modification causes a slight conformational modification which disrupts the binding of the DLG motif of Nrf2’s Neh2 domain, all while keeping the ETGE motif intact with Keap1 [49]. While ETGE binding is intact to Keap1, the DLG binding is much weaker, resulting in quick disassociation, fine-tuning ubiquitination of Nrf2 [50]. Upon this modification of a specific cysteine residue, Nrf2 escapes from Keap1, translocating to the nucleus and binding and inducing expression of Antioxidant response elements (AREs)-containing cytoprotective genes [51]. This initiates a transient, protective transcriptional response. Traditionally, it was thought that this Nrf2 signaling provided cancer chemoprevention [52]. However, in recent years, evidence has suggested that the cytoprotective function of Nrf2 might convey a survival benefit to cancer cells, thus suppressing the efficacy of the majority of presently developed chemotherapy.

Cancer cells, especially lung cancer, seek persistent activation of the Nrf2 pathway to build a permanent antioxidant shield [53]. This is achieved through somatic mutations, epigenomic errors, exon skipping, etc. However, the most direct mechanism is through somatic mutations that disrupt the Keap1-Nrf2 interaction [54,55]. The occurrence of the genetic mutations in Nrf2 is documented concerning Loss-of-function (LOF) somatic mutation in the KEAP1 gene, elevating levels of transcription activity in tumor cells, presenting survival benefits to the cancer cells [56]. LOF mutations are documented in lung, gallbladder, ovary, breast, liver, and stomach carcinomas [57]. Being a master regulator of oxidative stress response, Nrf2 sits at the center of a regulatory network that leads to the initiation and development of diseases like cancer [58]. The transcriptional program activated by Nrf2 is vast, encompassing hundreds of genes that collectively form the cellular antioxidant shield. Central to this Nrf2-driven defense is the machinery responsible for the synthesis, function, and recycling of GSH. As the cell’s most abundant non-protein thiol, the GSH system represents the primary pillar of the antioxidant response, and its fortification is a key consequence of constitutive Nrf2 activation in cancer.

#### 2.2.2. The Glutathione (GSH) System

As the most abundant non-protein thiol in the cell, the glutathione (GSH) molecule serves as the primary and most versatile intracellular antioxidant shield, especially for cancer cells [59]. GSH, an important regulator of redox cell signaling, is biosynthesized in a well-managed pathway. In which the three precursor amino acids, namely L-glutamate, cysteine, and glycine, are combined to form the tripeptide GSH [60]. It is synthesized mainly in the cytosol of hepatocytes and then imported into the mitochondria and nucleus in a continuous two-step enzymatic reaction, which is ATP-dependent [61]. The first reaction in the biosynthesis of GSH is a ligation reaction involving glutamate and cysteine to form γ-glutamylcysteine, catalyzed by glutamate-cysteine ligase (GCL), which is a rate-limiting step [62]. This dipeptide then combines with glycine by GSH synthetase (GSS) to finally produce Glutathione [63]. Crucially, the availability of cysteine governs the entire rate of GSH production. And cancer cells, addicted to this antioxidant shield of GSH, drive the upregulation of cysteine/glutamine antiporter, system Xc^−^ (encoded by the gene SLC7A11) [64], ensuring a continuous influx of crucial precursor, cystine, which is rapidly reduced to cysteine intracellularly, aiding in GSH production [65]. GSH acts by scavenging ROS directly in conjunction with enzymes, but its primary function is to serve as a co-substrate for powerful antioxidant enzymes. The most prominent of these are the cytosolic enzyme glutathione peroxidases (GPXs) belonging to the class of selenocysteine enzymes, and using GSH as a co-substrate to reduce H_2_O_2_ and other organic peroxides to harmless water and alcohols [66]. A critical member of this family, GPX4, is the sole enzyme capable of neutralizing phospholipid hydroperoxides (PLOOH) within cellular membranes and inhibiting microsomal lipid peroxidation, thereby acting as the master guardian against the iron-dependent regulated cell death pathway known as Ferroptosis [67,68]. Another key family, the Glutathione S-Transferases (GSTs), although they have presence in the membrane, mitochondria, and cytoplasm, in humans, the most diverse groups of GSTs are present as cytosolic enzymes, play a central role in the detoxification of reactive electrophile substances like carcinogens, mutagens, and tetragens [69]. GSTs catalyze the conjugation of GSH to a wide range of cytotoxic compounds, including many conventional chemotherapy agents, xenobiotics, and oxidative intermediates (DNA hydroperoxides and aldehydes), marking them for cellular efflux [70]. This GST-mediated drug clearance is a major mechanism contributing to acquired chemoresistance in cancer.

To maintain the antioxidant shield, the oxidized GSH-GSSG must be rapidly recycled back to GSH [71]. This vital task is performed by the enzyme Glutathione Reductase (GR), where nicotinamide adenine dinucleotide phosphate/H^+^ (NADPH/H^+^) acts as a crucial electron source [72]. To avail the benefit of this antioxidant system, cancer cells ensure a continuous supply of NADPH/H^+^ by upregulating the Pentose Phosphate Pathway (PPP), tightly coupling their metabolic state to their antioxidant capacity [73].

#### 2.2.3. The Thioredoxin (Trx) System

Complementing the glutathione system, the thioredoxin system—comprising thioredoxin (Trx), thioredoxin reductase (TrxR), thioredoxin-interacting protein, and NADPH- represents the second major antioxidant and redox-regulating hub in the cell. Its functions are diverse, ranging from scavenging ROS, transcription, DNA synthesis, cell growth stimulation, and repairing proteins that have been oxidatively damaged, thereby maintaining protein function and redox homeostasis [74].

The central player is Trx, a small 12 kDa redox protein containing a highly reactive dithiol active site that directly reduces oxidized cysteine residues on a vast number of target proteins [75]. In doing so, Trx itself undergoes oxidation. To complete the catalytic cycle, the oxidized Trx is reduced by the selenoenzyme Thioredoxin Reductase (TrxR), a reaction that is critically dependent on NADPH as the electron donor [74]. A major function of this system is to contribute to the peroxiredoxins (PRDXs), a family of highly abundant enzymes, by modulating the redox status, and act as primary sensors and efficient scavengers of H_2_O_2_ [76]. PRDXs are rapidly oxidized by H_2_O_2_, and their constant regeneration by Trx makes them a major peroxide-detoxifying pathway in the cell [77].

The Trx system is profoundly implicated in tumor biology and progression at different levels, and many cancer cells exhibit clear dependency on its function. Several studies indicated upregulation of Trx expression in different types of cancers, such as breast, gastric, lung, and pancreatic, directly correlating with cancer cell growth [78]. Txr acts by reversing the oxidation of key signaling proteins; it ensures the activation of pro-survival pathways [79]. Dysregulated proliferation is a hallmark of tumors, and cancer cells have a high demand for ongoing DNA synthesis to maintain rapid proliferation and expand tumor mass [80]. Critically, this demand is fulfilled by Thioredoxin (Trx), which serves as the obligate electron donor for ribonucleotide reductase, an enzyme essential for synthesizing the deoxynucleotides needed for DNA replication [81]. This indispensable role in both redox maintenance and proliferation makes the Trx system, particularly the enzyme TrxR, an attractive target for anticancer drug development [75,81]

## 3. Redox Regulation of Cancer Hallmarks

ROS, apart from being just indiscriminate agents of damage, function as highly specific signaling molecules in redox biology [82]. This signaling is primarily achieved through the reversible oxidation of critical cysteine (Cys) residues within target proteins, a post-translational modification that is central to redox biology [83]. Compared to other ROS, H_2_O_2_ exhibits low overall reactivity but is highly sensitive to the thiol group of cysteine residues and therefore proves to be the ideal ROS for this role [1,84]. The sensitivity of a specific cysteine is governed by its pKa, accessibility, and local microenvironment. When the finely tuned homeostasis is disrupted in cancer, sustained oxidative modification dysregulates the key signaling pathways that drive the acquisition of the malignant hallmarks.

### 3.1. Sustaining Proliferation and Evading Growth Suppressors via PTP Inactivation

A primary mechanism by which cancer cells achieve sustained proliferation is by dysregulating pro-proliferative signaling pathways. A paradigmatic example of this redox-driven dysregulation is the oxidative inhibition of protein tyrosine phosphatases (PTPs) [85]. PTPs are the critical enzymes that dephosphorylate tyrosine residues on target proteins, thereby signaling and counterbalancing signals from Protein Tyrosine Kinases (PTKs), such as Receptor Tyrosine Kinases (RTKs) [33,86]. Receptor tyrosine kinases (RTKs) regulate cellular signaling, which coordinates vital cellular processes such as proliferation, survival, growth, and metabolism upon interaction with growth factors and chemokines [87].

The PTP family is vast, comprising classical PTPs and 63 dual-specificity phosphatases (DSPs), showcasing their diversity and complexity [88], but many members critically depend on a highly reactive cysteine in their signature motif (I/V)HCXAGXGR(S/T) for the catalytic function. This cysteine is responsible for nucleophilic attack on the phosphotyrosine substrate to generate an intermediate phospho-cysteine. After this, the phosphate group is eliminated by hydrolysis via a conserved Asp residue within their WDP loop to reconstruct the native enzyme [89]. The low pKa of this cysteine, essential for its catalytic function, also makes it exceptionally susceptible to oxidation by ROS, which leads to its inactivation [90]. This oxidation converts the cysteine’s thiol (-SH) to a sulfenic acid (-SOH), inactivating the enzyme. While this is often reversible through the action of the thioredoxin system, further oxidation to irreversible sulfinic (-SO_2_H) or sulfonic (-SO_3_H) acids can permanently disable the phosphatase [91]. In the high-ROS environment of a cancer cell, this creates a deep imbalance. RTKs remain hyper-phosphorylated while their opposing PTPs are constantly shut down, leading to sustained pro-growth signaling [92]. This directly drives the hallmark of sustained proliferative signaling.

### 3.2. Evading Growth Suppressors via Oxidation and Inactivation of Tumor Suppressors Like PTEN and p53

This same mechanism allows cancer cells to evade growth suppressors, as key tumor suppressor phosphatases are prime targets of oxidative inactivation. The most notable example is PTEN (Phosphatase and Tensin Homolog). PTEN is a member of the PTP family, identified as a tumor-suppressor gene functioning via its lipid phosphatase activity, with a specific role in cell growth regulation [93]. The oxidative inactivation of PTEN has profound downstream consequences, most notably the hyperactivation of the PI3K/AKT/mTOR (PAM) pathway, a master regulator of cell growth, survival, and proliferation that is dysregulated in over half of human cancers [94,95]. PTEN is the primary brake on this pathway; its inactivation allows for the unchecked conversion of PIP2 to PIP3 by PI3K at the cell membrane, leading to the recruitment and constitutive activation of the kinase AKT [96]. Activated AKT then releases a cascade of pro-survival signals, principally through the mammalian target of rapamycin (mTOR). Operating via its mTORC1 and mTORC2 complexes, mTOR orchestrates the massive metabolic and biosynthetic programs required for tumor growth [97,98].

Beyond PTPs, ROS can also directly impact other major tumor suppressors. The redox-sensitive “guardian of the genome,” p53, which can act both pro- and anti-apoptotically, can be oxidatively modified on specific cysteine residues, inhibiting its sequence-specific DNA binding and activating its tumor-suppressive transcriptional program [99]. This provides a direct, redox-mediated mechanism for neutralizing two of the most important tumor suppressor pathways in human cancer.

The Mitogen-Activated Protein Kinase (MAPK) cascades are also intensely regulated by redox signaling, although the outcomes are highly context-dependent. The Mitogen-Activated Protein Kinase (MAPK) signaling cascades—comprising the ERK, JNK, and p38 subgroups- are activated upon oxidative stress by ROS [100]. The pro-proliferative ERK pathway is often potentiated by ROS through the oxidative inactivation of its endogenous negative regulators, the Dual-Specificity Phosphatases (DUSPs) [101]. The inactivation of ERK-specific phosphatases (e.g., DUSP6) prevents the dephosphorylation of ERK1/2, leading to a sustained signal for cell growth [102].

### 3.3. Resisting Cell Death

Cancer cells exhibit a profound ability to evade programmed cell death, a hallmark heavily influenced by intricate redox regulatory mechanisms. A primary pathway underpinning this resistance involves Nrf2-driven transcription. Constitutive activation of Nrf2 in cancer cells directly drives the transcriptional upregulation of crucial anti-apoptotic genes, notably BCL-2 and BCL-xL. This powerful antioxidant response elevates the threshold for apoptosis, rendering cancer cells less susceptible to intrinsic death signals and many therapeutic interventions [103]. Concomitantly, ROS-mediated activation of NF-κB also plays a significant role. Elevated ROS can activate the IKK complex, leading to the degradation of IκBα and the subsequent release of the NF-κB transcription factor (comprising subunits like p65/p50). Nuclear NF-κB then rewrites the expression of numerous pro-survival factors, including cIAP and XIAP, further contributing to an increased threshold for apoptosis and overall resistance to both endogenous death signals and conventional cancer therapies [104,105].

### 3.4. Inducing Angiogenesis

The sustained growth of tumors beyond a minimal size necessitates the formation of new blood vessels, a process termed angiogenesis, which is significantly regulated by the cellular redox state. A key redox-dependent mechanism driving angiogenesis is HIF-1α stabilization, often referred to as pseudohypoxia. Under conditions of elevated ROS, oxygen-sensing prolyl hydroxylase (PHD) enzymes become oxidatively inactivated, as ROS oxidizes their essential Fe (II) cofactor. This inactivation prevents the hydroxylation of HIF-1α, thereby blocking its VHL-mediated degradation [106]. Consequently, HIF-1α stabilizes and translocate to the nucleus even under normoxic conditions, where it heterodimerizes with ARNT. This complex then transcriptionally upregulates numerous pro-angiogenic factors, most notably vascular endothelial growth factor (VEGF). The constitutive transcription and secretion of these factors stimulate neovascularization, ensuring the growing tumor receives a vital supply of oxygen and nutrients [107].

### 3.5. Activating Invasion & Metastasis

Cancer’s metastatic cascade, encompassing invasion and distant dissemination, is profoundly influenced by redox signaling at multiple stages. One critical mechanism involves matrix remodeling, where ROS-mediated activation of matrix metalloproteinases (MMPs) plays a central role, often via the ‘cysteine switch’ mechanism. MMP-2 and MMP-9 are key molecular players in degrading the extracellular matrix, facilitating invasion [108]. Furthermore, ROS acts as a second messenger for pro-metastatic pathways like TGF-β, driving epithelial–mesenchymal transition (EMT) induction through the expression of EMT transcription factors, which confers migratory and invasive properties [109]. Lastly, anoikis resistance—the ability of detached cells to survive in circulation—is fortified by the Nrf2-driven antioxidant shield, which protects these circulating tumor cells from ROS-induced death, enabling their survival and subsequent metastatic seeding. Collectively, these redox-driven processes promote the degradation of the basement membrane, the acquisition of a migratory phenotype, and the survival of circulating tumor cells, all critical steps in metastatic spread [110].

### 3.6. Deregulating Cellular Metabolism

The metabolic reprogramming characteristic of cancer, particularly the Warburg Effect, is intricately linked to redox homeostasis. Enzymatic and transcriptional control by ROS directly contributes to this deregulation. ROS can directly oxidize and modulate the activity of key metabolic enzymes, for instance, inhibiting pyruvate kinase M2 (PKM2) to divert glucose flux towards the pentose phosphate pathway. This metabolic shift provides biosynthetic precursors crucial for rapid cell proliferation and generates NADPH, an essential cofactor for antioxidant defense [111]. Concurrently, ROS-stabilized HIF-1α, transcriptionally upregulates glycolytic enzymes, further promoting the Warburg Effect. This metabolic adaptation favors the production of essential biosynthetic precursors and NADPH over efficient ATP generation, providing the building blocks and redox buffering capacity required for sustained tumor growth [112].

### 3.7. Genome Instability & Mutation

A hallmark fundamental to cancer progression and evolution is genome instability and mutation, a process significantly exacerbated by persistent oxidative stress. The hydroxyl radical (•OH), a highly reactive ROS, can directly oxidize DNA bases, creating mutagenic lesions such as 8-oxoG. These DNA adducts are potent mutagens that can lead to mispairing during replication [113]. Simultaneously, chronic ROS can impair the function of crucial DNA repair enzymes, preventing the effective correction of these lesions. The interplay of direct DNA damage and compromised repair mechanisms leads to an increased somatic mutation rate and chromosomal instability [114]. This genomic instability fuels tumor progression, raises intra-tumoral heterogeneity, and significantly contributes to the development of drug resistance, driving the aggressive phenotype of many cancers. Table 1 provides a comprehensive overview of this pervasive influence, the key redox-dependent mechanisms, molecular players, and functional outcomes for each major hallmark.

## 4. The Tumor Microenvironment (TME)

While the intracellular redox reprogramming of cancer cells is foundational to their survival, the story of malignancy is incomplete without expanding the view to the surrounding tumor microenvironment (TME). A tumor is an organ of cancer cells, and is not merely a group of cells. It rather consists of heterogeneous infiltrating and resident host cells, secretory factors, and extracellular matrix, together called the TME [115]. The TME has been shown to play a pivotal role in tumor initiation, development, and metastasis, actively manipulating the redox state of its neighbors [116]. This corruption transforms them into co-conspirators that support tumor growth, provide metabolic fuel, and suppress anti-tumor immunity. It is critical to note that within this ecosystem, redox signaling emerges as the primary language of intercellular crosstalk. Various cells and factors play a role in the TME, namely, immune cells, natural killer (NK) cells, extracellular matrix (ECM), etc. [117]. And amongst these cells, fibroblasts have been suggested to play a key role [118].

### 4.1. Cancer-Associated Fibroblasts (CAFs)

Normal fibroblast represents the majority of the stromal cells, and in the initial phase of tumor development, their role is inhibitory, acting via simple gap junctions between fibroblasts and IL-6 [119]. However, they are re-educated by signals from cancer cells, including persistent oxidative stress, to adopt and transform into a pro-tumorigenic cancer-associated fibroblast (CAF) phenotype [120]. These activated fibroblast cells are particularly identified by expression of different biomarkers, such as smooth muscle actin, vimentin, desmin, and fibroblast activation protein [121]. The CAF participates in the malignancy by actively secreting cytokines and chemokines, such as vascular endothelial growth factor A (VEGFA) and C-X-C motif chemokine ligand 12(CXCL12) [122]. Tumors are known to exhibit profound deviations from conventional biological processes, particularly evident in the highly reprogrammed metabolism of cancer cells [123].

The metabolic corruption within the TME gives rise to a sophisticated strategy: the ‘Reverse Warburg Effect’. In this model, the cancer cell, benefiting from ROS production, floods the TME with H_2_O_2_. This oxidative stress, coupled with the loss of key regulators like caveolin-1 in the fibroblasts, drives the stabilization of HIF-1α [124]. The CAFs, following metabolic reprogramming, dramatically increase their rate of aerobic glycolysis, breaking down glucose into high-energy metabolites like lactate and pyruvate, which are transported from CAF to TME via monocarboxylate transporter 4 (MCT4). This secreted lactate is a high-potency fuel source efficiently imported via monocarboxylate transporter 1 (MCT1) [125]. Once inside the cancer cell, the lactate is converted back to pyruvate and shuttled into mitochondria to oxidatively metabolize these energy-rich metabolites via the TCA cycle and OXPHOS to produce large quantities of ATP from cancer cells [126]. Standard glycolysis alone is inefficient in providing such a massive amount of ATP; these enormous energy reserves allow cancer cells to proliferate, invade, and develop therapy resistance.

### 4.2. Immune Cells

Beyond metabolic reprogramming of fibroblasts, redox signaling plays an equally crucial, if not more insidious, role in shaping the immune landscape of the tumor microenvironment. A fundamental requirement for tumor survival is the evasion of immune destruction, a hallmark achieved by creating a profoundly immunosuppressive local environment. Cancer cells orchestrate this by weaponizing ROS and RNS, using them not only for intracellular signaling but as paracrine agents to directly neutralize, exhaust, and suppress anti-tumor immune cells.

Central to the creation of an immunosuppressive TME are two key myeloid populations: Myeloid-Derived Suppressor Cells (MDSCs) and Tumor-Associated Macrophages (TAMs). Rather than attacking the tumor, these cells are co-opted and reprogrammed by cancer cells to promote disease progression [127], act as local enforcers, actively suppressing the immune function of T-cells and causing changes in B-cells in a contact-dependent way [128]. They achieve this primarily through the enzymatic production of high levels of ROS and RNS [129]. Upon recruitment to the tumor, MDSCs and TAMs upregulate the expression of NOX2 and iNOS. The resulting flux of superoxide and NO has profound inhibitory effects on effector T-cells. For instance, these reactive species can nitrate the T-cell receptor (TCR), rendering it unable to recognize its antigen [130], and can also lead to the depletion of L-arginine, an amino acid essential for T-cell proliferation and function. This way, MDSCs and TAMs use redox weaponry to neutralize the body’s primary anticancer defense.

## 5. Therapeutic Strategies Targeting Redox Vulnerabilities in Cancer

The extensive redox reprogramming described in the preceding sections, while a key driver of malignancy, paradoxically creates a unique and exploitable therapeutic vulnerability [131]. Unlike normal cells, which maintain low basal ROS levels and a large capacity to buffer oxidative damage, cancer cells exist in a state of chronic oxidative stress, operating perilously close to a toxic threshold beyond which cell death is inevitable [132]. Their survival is entirely dependent on their hyperactive antioxidant machinery (e.g., the Nrf2, GSH, and Trx systems). Cancer cells are ‘addicted’ to high ROS for signaling, but exquisitely sensitive to further increases, creating a heightened therapeutic window [133]. It allows for the development of strategies that can selectively target cancer cells by modulating their redox state.

### 5.1. Therapeutic ROS Induction

Conventional Therapies

Tumor radiotherapy or radiation therapy (RT) is a conventional technique used to inhibit and control the growth, proliferation, and even metastasis of the malignant tumor cells via various types of ionizing radiation. It is a primary cancer treatment modality, and the cancer cures account for at least 40% [134]. Radiation therapy (RT), which includes external beam radiation, brachytherapy, and radioisotope therapy, is often provided alone for localized tumors [135]. Although primarily delivered via external beam radiation therapy, brachytherapy, and radioisotope therapy for localized tumors, radiation therapy has seen many advances in recent years [136].

Radiation therapies bring changes in the biological properties of the cancer cells, and the major effects on tumor tissues are apoptosis, necrosis, and senescence induced by DNA damage [137,138]. During radiotherapy, a massive burst of highly reactive ROS, most notably the hydroxyl radical (•OH), is generated [137]. These radicals indiscriminately attack cellular macromolecules, damaging tumor cell DNA, forming lesions, opening the deoxyribose ring, and causing single and double-stranded breaks (DBS) [139]. For cancer cells that are already operating at a high basal level of oxidative stress, this sudden, immense wave of ROS is sufficient to overwhelm their antioxidant defenses, pushing them past the toxic threshold and leading to cell death; therefore, targeting DNA damage is a promising approach for therapy [140]. Similarly, many classic chemotherapeutic agents, such as platinum-based drugs (e.g., cisplatin) and anthracyclines (e.g., doxorubicin), also exert a significant portion of their cytotoxic effects by disrupting mitochondrial function and promoting massive ROS production [141].

Targeted Pro-Oxidant Drugs

Building on the principle of ROS-induced cytotoxicity, a new generation of drugs has been developed to specifically and selectively elevate oxidative stress within cancer cells. A prominent, if once controversial, example is high-dose vitamin C (ascorbate). While dietary vitamin C functions as a classic antioxidant [142], the pharmacological concentrations achievable only through intravenous administration cause it to act as a potent, tumor-selective pro-oxidant [143]. Vitamin C at a concentration ranging from 0.25–2.0 mM induces significant apoptosis in AML cell lines, as reported in an original study of 2013 [144]. The mechanism hinges on the Fenton reaction. In the iron-rich tumor microenvironment, ascorbate reduces ferric iron (Fe^3+^) to ferrous iron (Fe^2+^), which then reacts with O_2_ to produce a substantial flux of H_2_O_2_ [145]. This creates a therapeutic window because, compared to normal cells, many cancer cells are deficient in H_2_O_2_-detoxifying enzymes, particularly catalase [146]. This relative catalase deficiency allows H_2_O_2_ to accumulate to cytotoxic levels, inducing oxidative damage and cell death [147]. Indeed, numerous studies have demonstrated this effect, with millimolar concentrations of ascorbate inducing apoptosis in AML and breast cancer cell lines [148,149]. Consequently, high-dose intravenous ascorbate is now being rigorously investigated in clinical trials, typically in combination with standard chemotherapy or radiation, as a strategy to selectively poison cancer cells with ROS [150].

Another powerful clinical example of a pro-oxidant therapy is Arsenic Trioxide (ATO), a compound that has transformed the treatment of Acute Promyelocytic Leukemia (APL) and is approved by the U.S. Food and Drug Administration for use [151,152]. A study reported doses ranging from 1–300 μM of ATO induce massive ROS production via mitochondria, initiating structural changes in DNA, including base-pair mutations, translocation, deletion, as well as DNA hype and hypomethylation [151]. In another study, promyelocytic leukemia (PML), ATO showed high binding affinity to cysteine residues, resulting in the initiation of PML/RARα (retinoic acid receptor α) gene degradation [153]. One separate study also showed that the ATO can induce inhibition of cell growth and apoptosis at 1–2 μM concentration, also in solid tumors such as colon cancer and neuroblastoma [154]. In case of lung cancer, ATO at low doses 1, 2, and 4 μM has shown inhibition and cell cycle arrest [155].

A third, highly promising pro-oxidant strategy is represented by Piperlongumine (PL), a biologically active alkaloid isolated from the long pepper plant (Piper longum) [156]. In recent years, PL showed apoptotic cell death in bladder, colon, breast, pancreatic, osteosarcoma, and lung cancer cells [157]. It exhibits remarkable selectivity for cancer cells over normal cells; a specificity derived from its unique mechanism of action. Functioning as a ROS-activated pro-drug, it modulates and or inhibits redox enzymes essential for redox homeostasis [158].

### 5.2. Targeting Antioxidant Capacity

In a conceptually opposite yet complementary approach, the second major strategy for exploiting redox vulnerabilities involves disrupting the cancer cell’s intrinsic antioxidant shield. As established previously, cancer cells are addicted to their hyperactive antioxidant systems—the Nrf2, glutathione, and thioredoxin pathways—to survive high levels of endogenous ROS [159]. By specifically inhibiting this antioxidant system, it is possible to trigger a catastrophic rise in oxidative stress, leading to selective cancer cell death. Furthermore, these inhibitors hold potential as chemo- and radio-sensitizers, capable of weakening a tumor’s defenses and making them vulnerable to the effects of conventional therapies.

Nrf2 Inhibitors

As discussed earlier, transient activation of Nrf2 exhibits protective, anti-carcinogenic, and anti-mutagenic activities on non-malignant cells [160], but constitutive activation promotes therapeutic resistance and aggressive tumorigenic ability, driving malignant progression in cancer cells [161]. Hence, permanent inhibition of the Nrf2 pathway shows therapeutic potential. One such promising drug for this role is Brusatol. It is a quassinoid compound with various pharmacological effects, mainly anti-inflammatory and anti-tumor activity, suppressing the Nrf2 signaling pathway [162]. In past studies, it has shown tumor growth inhibition in pancreatic, colorectal, hepatocellular, and non-small cell lung cancer [163]. Brusatol indirectly inhibits Nrf2 by inhibiting global protein translation and additionally through rapid and transient depletion of Nrf2 protein through a posttranscriptional mechanism in mouse Hepa-1c1c7 hepatoma cells [164]. At a concentration of 20–40 nM in a cell culture line or in mice intraperitoneal administration at a dose range of 1–2 mg/kg, brusatol showed efficient Nrf2 protein reduction [45].

One main challenge with this drug acknowledged across studies remains its specificity. Brusatol is proven to be a potent Nrf2 inhibitor, but several studies report different mechanisms; hence specificity of the drug remains unclear [165]. Additionally, a specific small molecule inhibitor of Nrf2, ML385, specifically and directly interacts with the Nrf2 protein, blocking its transcriptional activity and marked tumor growth inhibition [166]. ML385 has also proven to inhibit cancer cell growth when administered in conjunction with an anti-tumor natural compound, Celastrol [167].

GSH & System Xc-Inhibitor

Given its role as the primary antioxidant buffer, the glutathione system is a logical and extensively studied target. The classic approach to depleting cellular GSH involves inhibiting its synthesis directly [168]. Buthionine sulfoximine (BSO) is an irreversible inhibitor of γ-glutamyl cysteine synthetase (γ-GCS), the rate-limiting enzyme in GSH biosynthesis [61]. In a study of BSO conjugated on hollow gold nanoparticles produced a dramatic loss of intracellular GSH levels in A549 cells in human lung cancer [169]. Although BSO showed depleted GSH in the tumor, substantial therapeutic benefits were minimal; hence, with BSO therapy, sensitive cancer patients selected using sensitivity markers might provide more clinical efficacy [170]. While showing limited efficacy as a monotherapy, BSO has been tested extensively in clinical trials as a potent chemo- and radio-sensitizer, demonstrating the principle of weakening the shield to enhance conventional therapies [171].

A more modern strategy focuses on starving the cell of cysteine, the essential precursor for GSH synthesis. This is achieved by inhibiting the System xc^−^ (SLC7A11) cystine/glutamate antiporter [172]. The tool compound Erastin and the repurposed FDA-approved drug Sulfasalazine (SSZ) are well-known inhibitors of this transporter. Studies have shown that SSZ decreases GSH levels and increases ROS accumulation by inhibiting xCT [64,173]. One study reported Erastin inhibits cystine uptake efficiently with an IC_50_ value of around 1.4 µM [174]. This approach is particularly powerful as it delivers a dual blow: it depletes the GSH pool and, as previously discussed, is a potent inducer of ferroptosis, a non-apoptotic cell death pathway driven by lipid peroxidation [64,175]. This dual mechanism makes System xc^−^ inhibitors a highly attractive therapeutic avenue.

Trx System Inhibitor

The thioredoxin system, with its central role in protein repair and DNA synthesis, represents another critical node for therapeutic intervention [176]. A prime example of targeting this pathway is the repurposing of the drug Auranofin. Originally an FDA-approved gold-containing compound for treating rheumatoid arthritis, Auranofin was later discovered to be a potent and irreversible inhibitor of thioredoxin reductase (TrxR) [177]. By disabling TrxR, AF prevents the regeneration of active thioredoxin, leading to an accumulation of oxidized proteins and a dramatic increase in intracellular ROS [178]. This mechanism has proven effective in preclinical models of various cancers, particularly ovarian cancer and certain leukemias, and AF is being actively investigated in oncology clinical trials. It was demonstrated that AF could induce cell death in various breast cancer cell lines with IC_50_ values ranging between 0.5 and 2 µM in TNBC cells and impaired the growth of TNBC-derived spheroids [179]. Another study observed AF inhibits the NSCLS cell line with an IC_50_ value of less than 1.0 µM [180]. In vitro evidence showed that in CRC, AF analogs exhibit cytotoxic effects [181].

### 5.3. Redox-Active Metal Complexes Therapeutics

While cancer cells adeptly recalibrate their endogenous antioxidant defenses to survive elevated intrinsic reactive oxygen species (ROS), this delicate balance also exposes crucial vulnerabilities that could be therapeutically exploited by exogenous agents [182]. Among the most sophisticated classes of such compounds are the redox-active metal complexes, particularly Metalloporphyrins (MnPs), and Manganese (II) (Mn (II)) pentaaza macrocyclic compounds, originally developed as mimics of SOD. They are designed to strategically manipulate cellular redox homeostasis by interacting with numerous reactive species [183]. Historically, the development of these compounds began with the search for potent superoxide dismutase (SOD) mimics to alleviate general oxidative stress, with cationic Mn (III) *N*-substituted pyridylporphyrins [Mn (III) porphyrins] demonstrating remarkable O_2_^•−^ dismutation capabilities, comparable to native SOD enzymes [184]. However, recent advances have elucidated their more nuanced and context-dependent roles, revealing them as powerful agents capable of both pro-oxidant and indirect Nrf2-mediated antioxidative mechanisms [184].

In healthy tissues, where basal ROS levels are typically lower, these compounds often exert an apparent antioxidant effect, a characteristic crucial for their therapeutic window [185]. However, the current mechanistic understanding emphasizes that this protection is largely indirect, stemming from their intricate pro-oxidative interactions that modulate key cellular signaling pathways, rather than solely direct scavenging of reactive oxygen species [186]. While Mn porphyrins can efficiently reduce or disproportionate species such as ONOO^−^ or O_2_•^−^ upon direct encounter, this is generally not considered their major mode of action for protective effects [184].

Instead, in normal cells, Mn porphyrins predominantly operate via two distinct, yet interconnected, pathways. Firstly, they engage in the oxidation of the NF-κB subunit p65, which leads to the inhibition of its transcription and, consequently, the suppression of proinflammatory processes. Secondly, and critically, they modulate Nrf2 pathway activity. Rather than acting as a direct superoxide dismutase (SOD) mimic, Mn porphyrins exert a pro-oxidative effect by catalyzing the oxidation of Keap1 cysteines. This action releases Nrf2, allowing it to translocate to the nucleus, where its binding to AREs results in the transcription and subsequent upregulation of the cell’s own endogenous antioxidative defenses, such as MnSOD and catalase. This indirect mechanism has been demonstrated in normal hematopoietic progenitor cells [187]. Furthermore, historical data from the Thambi Dorai group, which showed a Mn hexyl analog upregulating several endogenous antioxidative defenses, is now reinterpreted in light of these recent insights into MnP-driven Nrf2 activation [188]. This sophisticated, indirect redox modulation, particularly via Nrf2 activation, ultimately underpins their promise in radioprotection, safeguarding normal cells from radiation-induced damage [189].

Key examples include Manganese (III) (Mn (III)) porphyrins such as MnTE-2-PyP (BMX-010) and, notably, MnTnBuOE-2-PyP^5+^ (BMX-001), which has advanced significantly in clinical development [190]. Another prominent example is M40403/GC4419 (avasopasem manganese), a compound significantly influenced by researchers like Doug Spitz and his colleagues [191]. It is important to note, however, that the mechanism of action for Mn cyclic polyamines like GC4419/M40403 may differ from that of Mn porphyrins. Research by Batinic-Haberle et al. suggests that compounds like M40403 primarily increase H_2_O_2_ levels through superoxide dismutation, thereby acting more directly as a superoxide dismutase (SOD) mimic [184].

This pro-oxidant shift is critically enhanced when combined with conventional therapies like chemo- and radiotherapy, or adjuvant agents like ascorbate, amplifying cytotoxicity by leveraging existing high oxidative stress in cancer cells [192]. At a molecular level, compounds like BMX-001 excel at catalyzing the H_2_O_2_-driven oxidation of key signaling proteins, such as NF-κB, in aggressive ovarian cancer cell lines, thereby disrupting pro-survival pathways and leveraging the tumor’s intrinsic redox imbalance. A significant therapeutic advantage also lies in their ability to modulate the Nrf2 pathway. While Nrf2 activation often confers resistance to cancer cells, recent findings demonstrate that BMX-001, combined with paclitaxel (PTX), effectively suppresses Nrf2 activity in high-grade ovarian cancer. This crucial ability offers a powerful strategy to overcome drug resistance and enhance combination therapy efficacy [193]. Indeed, BMX-001 was, for the first time, shown to suppress the growth of these very aggressive high-grade serous ovarian cancer cells with high oxidative stress as efficaciously as PTX in its own right, thereby demonstrating its potent standalone anticancer activity without the explicit need for combination with chemo- or radiotherapy [194,195]. The clinical translation of these findings is increasingly evident. BMX-001 has advanced into five distinct Phase II clinical trials for various cancers, reflecting its broad potential [196,197].

In conclusion, redox-active metal complexes like BMX-001 and GC4419 represent a highly promising frontier. Their ability to dynamically shift between antioxidant and pro-oxidant roles, leveraging differential oxidative stress and H_2_O_2_ levels, perfectly embodies the Redox Paradox. To provide a concise overview of these diverse therapeutic strategies and their current clinical development status, Table 2 summarizes the key approaches, representative agents.

## 6. Emerging Frontiers and Grand Challenges

One of the most exciting frontiers in redox-targeted therapy is the exploitation of ferroptosis, a non-apoptotic, iron-dependent form of regulated cell death first described in 2012 [198]. Ferroptosis is unique from other types of programmed cell death, depending on ROS derived from iron metabolism, lipid peroxidation, and ATP production for its induction [199]. It is long known that in comparison to non-malignant cells, malignant cells have a high demand for iron, allowing cancer cells to grow and proliferate rapidly [200,201]. A reaction between ferrous (Fe^2+^) or ferric (Fe^3+^) ions and H_2_O_2_ results in the Fenton reaction [202]. The hydroxyl radicals from this reaction trigger peroxidation of PUFA in membrane lipids, causing lethal accumulation of lipid hydroperoxide (L-OOH) products in the cell, a classical hallmark of ferroptosis, disrupting and breaking down the cell skeleton [203]. The primary defense against this process is GPX4. This places the GSH-System xc^−^-GPX4 axis as the master regulator of ferroptotic cell death [204]. GSH is the key component of GPX4, synthesized from cysteine molecules [199]. Cysteine is transported into cells via System Xc- by exchange of glutamate at a 1:1 ratio. This antiporter system and the cysteine molecule serve as the backbone of GSH synthesis [205]. This discovery has unveiled a powerful therapeutic strategy: inducing ferroptosis in cancer cells, particularly those that have become resistant to traditional apoptosis-based therapies [206]. This can be achieved either by directly inhibiting GPX4 or by depleting its essential cofactor, GSH, by blocking cysteine import via the System xc^−^ antiporter [207,208].

Two grand challenges currently limit the broad clinical application of redox-based therapies: acquired resistance and lack of specificity. The same Nrf2-driven antioxidant reprogramming that promotes tumorigenesis is also a primary mechanism by which cancer cells become resistant to conventional therapies that rely on ROS for their efficacy [209]. Overcoming this requires a shift towards precision medicine, driven by the development of predictive biomarkers [210]. Characterizing a tumor’s specific redox signature—through genetic sequencing of KEAP1/NRF2, immunohistochemistry for antioxidant proteins, or advanced imaging probes that can non-invasively map redox states—is essential for selecting the right patients for the right therapy [211,212,213]. Recent advancements, such as single-cell profiling in mouse carcinoma models, are providing unparalleled resolution in understanding tumor redox heterogeneity and its impact on therapeutic response. For instance, a detailed study utilizing single-cell profiling has revealed immune-based mechanisms underlying tumor radiosensitization by a novel Mn porphyrin clinical candidate, BMX-001, offering new avenues for precision redox medicine [192].

Furthermore, achieving tumor specificity to avoid systemic toxicity remains a major hurdle [214]. Here, nanotechnology offers immense promise, enabling the targeted delivery of redox-modulating agents specifically to cancer cells via passive accumulation (the EPR effect) or active targeting of tumor-specific surface receptors [215].

The unprecedented success of immune checkpoint inhibitors (ICIs) has been tempered by the reality that many patients do not respond, often due to a highly immunosuppressive tumor microenvironment [216,217]. Emerging evidence now positions the redox state of the TME as a critical determinant of this resistance and, therefore, as a prime target for combination therapy. As discussed previously, the high-ROS environment within a tumor is profoundly immunosuppressive [218]. It directly impairs the function and survival of cytotoxic T-lymphocytes and empowers myeloid-derived suppressor cells (MDSCs) to further inhibit the anti-tumor response [219,220]. The oxidative shield effectively renders T-cells exhausted and dysfunctional, making them unable to mount an effective attack even when the PD-1/PD-L1 brake is released by ICIs. This provides a strong rationale for a new therapeutic paradigm: combining ICIs with redox-modulating agents to create a more permissive environment for a successful anti-tumor immune response [221,222].

## 7. Conclusions

The intricate relationship between cancer and cellular redox state is not one of mere stress, but of purposeful adaptation. As this review has detailed, cancer’s grasp of redox biology represents a core dependency, fundamental to its survival and progression. Malignant cells systematically rewire their internal environment to maintain a heightened yet tightly controlled level of reactive oxygen species. This ‘redox reset’ is not a passive byproduct of transformation but an actively maintained state, where ROS is co-opted from a damaging agent into a critical signaling molecule that drives the acquisition of nearly every cancer hallmark, from unchecked proliferation to the exploitation of the tumor microenvironment. This deep-seated dependency, however, exposes a unique therapeutic vulnerability. The success of future redox-based cancer therapies will hinge on the ability to move beyond blunt, non-specific strategies and embrace a paradigm of Redox Precision Medicine. The development and clinical implementation of predictive biomarkers—from tissue-based analysis of Nrf2 activity to non-invasive imaging of a tumor’s specific redox signature—are therefore paramount. Ultimately, cracking the cancer cell’s ‘redox code’ is one of the great challenges and opportunities in modern oncology. The frontiers are clear: combining redox-modulating agents to reverse therapy resistance and synergizing them with immunotherapy to transform immunosuppressive microenvironments. The goal is to turn cancer cells’ most essential survival strategy into its catastrophic failure, exposing a new generation of personalized and effective cancer treatments.

## Figures and Tables

**Table 1:** Redox Regulation of the Hallmarks of Cancer.

	Key Redox-Dependent Mechanism(s)	Key Molecular Players	Downstream Consequences	References
3.1 Sustaining Proliferation	H_2_O_2_-mediated oxidative inactivation of catalytic cysteines in Protein Tyrosine Phosphatases (PTPs), thereby sustaining pro-growth signaling.	ROS (H_2_O_2_), PTPs (catalytic cysteine), Receptor Tyrosine Kinases (RTKs), Thioredoxin system (for reversibility).	Hyper-phosphorylated RTKs drive sustained pro-growth signaling and uncontrolled cell division due to inhibited PTP counteraction.	[86]
3.2 Evading Growth Suppressors	Oxidative inactivation of tumor suppressor phosphatases (e.g., PTEN) and direct oxidative modification of tumor suppressors (e.g., p53). Concurrently, ROS-mediated potentiation of pro-proliferative MAPK pathways (e.g., ERK) via DUSP inactivation.	ROS (H_2_O_2_), PTEN, PI3K/AKT/mTOR pathway, p53 (cysteine residues), Mitogen-Activated Protein Kinases (MAPKs: ERK, JNK, p38), DUSPs (e.g., DUSP6).	Hyperactivation of the PI3K/AKT/mTOR pathway leads to unchecked cell growth, survival, and proliferation; concurrently, p53’s tumor-suppressive functions are inhibited, and pro-growth MAPK signaling is sustained	[98]
3.3 Resisting Cell Death	1. Constitutive Nrf2 activation drives direct transcriptional upregulation of anti-apoptotic genes (e.g., BCL-2, BCL-xL). 2. ROS activates NF-κB (via IKK complex), orchestrating expression of pro-survival factors (e.g., cIAP, XIAP)	1. Nrf2, BCL-2, BCL-xL 2. ROS, IKKβ, IκBα, NF-κB (p65/p50), cIAP, XIAP	Increased threshold for apoptosis and expression of pro-survival factors, resulting in resistance to both endogenous death signals and cancer therapies.	[103,104,105]
3.4 Inducing Angiogenesis	ROS oxidizes the Fe (II) cofactor in prolyl hydroxylase (PHD) enzymes, inactivating them and blocking VHL-mediated HIF-1α degradation, leading to its stabilization (pseudohypoxia) and upregulation of pro-angiogenic factors (e.g., VEGF)	ROS (H_2_O_2_), PHDs, VHL, HIF-1α, ARNT, VEGF	Constitutive transcription and secretion of pro-angiogenic factors (e.g., VEGF), stimulating neovascularization to supply the growing tumor with oxygen and nutrients.	[106,107]
3.5 Activating Invasion & Metastasis	1. ROS-mediated activation of Matrix Metalloproteinases (MMPs) (e.g., via ‘cysteine switch’ 2. ROS as a second messenger for pro-metastatic pathways like TGF-β, driving EMT induction. 3. Nrf2-driven antioxidant shield fortifies anoikis resistance, protecting circulating tumor cells from ROS-induced death	ROS, MMP-2, MMP-9 TGF-β, Nrf2	Degradation of the basement membrane, acquisition of a migratory phenotype, and survival of circulating tumor cells collectively promote metastatic spread.	[108,109,110]
3.6 Deregulating Cellular Metabolism	ROS directly oxidizes and modulates key metabolic enzymes (e.g., inhibiting PKM2 to divert flux to the PPP). Concurrently, ROS-stabilized HIF-1α transcriptionally upregulates glycolytic enzymes, promoting the Warburg Effect.	ROS, PKM2, HIF-1α, Glycolytic enzymes	Promotion of the Warburg Effect, favoring the production of biosynthetic precursors (for new cells) and NADPH (for antioxidant defense) over efficient ATP generation.	[111]
3.7 Genome Instability & Mutation	Direct hydroxyl radical (•OH)-mediated oxidation of DNA bases, creating mutagenic lesions (e.g., 8-oxoG). Concurrently, ROS impairs the function of DNA repair enzymes, preventing lesion correction	ROS (•OH), 8-oxoG, DNA bases, DNA repair enzymes	Increased somatic mutation rate and chromosomal fueling tumor evolution, intra-tumoral heterogeneity, and the development of drug resistance.	[113]

**Table 2:** Summary of Therapeutic Strategies Targeting Redox Vulnerabilities in Cancer.

Therapeutic Approach/Category	Key Redox-Focused Mechanism(s)	Examples
Conventional Therapies	Induce massive ROS burst, causing DNA damage and overwhelming antioxidant defenses. Disrupt mitochondrial function; promote ROS production.	Radiation Therapy (RT) Chemotherapy (e.g., Cisplatin, Doxorubicin)
Targeted Pro-oxidant Drugs	Generate cytotoxic H_2_O_2_ or other ROS by leveraging tumor-specific redox features (e.g., high Fe, catalase deficiency). Induces mitochondrial ROS, DNA damage, and promotes PML/RARα degradation. ROS-activated pro-drug; modulates/inhibits redox enzymes.	High-dose Vitamin C (Ascorbate) Arsenic Trioxide (ATO) Piperlongumine (PL)
Nrf2 Inhibitors	Suppress Nrf2 pathway, weakening antioxidant defenses and sensitizing cancer cells to oxidative stress.	Brusatol, ML385
GSH & System Xc- Inhibitors	Inhibit GSH synthesis (BSO) or cysteine uptake (Erastin, SAS), depleting GSH and inducing oxidative stress/ferroptosis.	Buthionine sulfoximine (BSO) Erastin, Sulfasalazine (SAS)
Trx System Inhibitors	Inhibit Thioredoxin Reductase (TrxR), preventing Trx regeneration, increasing ROS, and protein oxidation.	Auranofin
Redox-Active Metal Complexes	Dual-Action: SOD mimics/radioprotectors in normal cells; pro-oxidants in cancer (H_2_O_2_-driven oxidation, Nrf2 suppression).	BMX-001 (MnTnBuOE-2-PyP) GC4419 (M40403) (Avasopasem manganese) AEOL10150 (MnTDE-2-ImP5+)

## Data Availability

Data sharing is not applicable.
